# Assessment of Network Integrity in Right-Hemispheric Glioma Patients Using Function-Based Tractography and Domain-Specific Cognitive Testing

**DOI:** 10.3390/cancers17244007

**Published:** 2025-12-16

**Authors:** Maximilian Schwendner, Leonie Kram, Johanna Lackner, Haosu Zhang, Sandro M. Krieg, Sebastian Ille

**Affiliations:** Department of Neurosurgery, Heidelberg University Hospital, Ruprecht-Karls-University Heidelberg, 69120 Heidelberg, Germany; maximilian.schwendner@med.uni-heidelberg.de (M.S.); leonie.kram@med.uni-heidelberg.de (L.K.); johanna.llackner@gmail.com (J.L.); haosu.zhang@gmail.com (H.Z.); sandro.krieg@med.uni-heidelberg.de (S.M.K.)

**Keywords:** neurocognitive testing, eloquent glioma, function-guided surgery, brain mapping

## Abstract

Objective: Gliomas disrupt functional brain networks and impair neurological functions. While left-hemispheric tumors are well-studied because of their impact on language domains, the influence of right-sided gliomas on higher cognitive functions remains less understood. This study aimed to assess pre- and postoperative neurocognitive performance and to link cognitive outcomes with structural findings derived from function-based tractography in patients with right-hemispheric gliomas. Methods: Patients with gliomas were enrolled in this prospective observational study. A structured neurocognitive test battery was administered preoperatively, postoperatively, and at 3-month follow-up. Preoperative cortical mapping using navigated transcranial magnetic stimulation (nTMS) and function-based fiber tracking, based on diffusion tensor imaging (DTI), was performed. Results: Eighteen patients aged 52.7 ± 18.3 years were included. Preoperatively, 88.8% of patients showed impairments in at least one cognitive test, most frequently in the Nine-Hole Peg Test (66.7%), Bells Test task completion time (61.1%), Trail Making Test A and B (TMT-A: 50.0%; TMT-B: 44.4%), and digit symbol substitution test (27.8%). At follow-up, task performance improved on most cognitive tests. Function-based tractography showed that involvement of the superior longitudinal fasciculi I–III (44.4% of cases) was associated with impairments in attention, executive function, visuospatial processing, and processing speed. The involvement of the inferior frontooccipital fasciculus (55.5% of cases) was related to deficits in processing speed, attention, executive function, and episodic memory. Conclusions: Neurocognitive deficits are common in patients with right-hemispheric gliomas even before surgery. Maximal safe resection and sparing of these tracts is associated with cognitive recovery at follow-up. Function-based tractography emphasizes the structural involvement of key association fibers related to these cognitive deficits.

## Simple Summary

Brain tumors can affect not only movement or language but also many subtle aspects of thinking, such as attention, memory, and coordination. These changes often remain unnoticed in daily clinical care, especially in patients with tumors on the right side of the brain. In this study, we tested patients with right-sided brain tumors before and after surgery using structured cognitive tasks and advanced imaging techniques to trace important brain connections and networks. We found that many patients showed difficulties in attention, processing speed, and coordination, which improved in some cases after surgery. The affected brain networks were mainly those connecting frontal and parietal brain regions. Understanding how right-sided tumors alter these brain networks helps surgeons plan operations more safely and may improve recovery and quality of life for patients after treatment.

## 1. Introduction

The concept of eloquent tumor location traditionally refers to brain regions whose injury leads to significant functional deficits. Accordingly, gliomas located within or adjacent to the primary motor cortex or left hemispheric language areas are often classified as eloquent. However, this approach may overlook the broader spectrum of cognitive functions that can be compromised in patients with gliomas [1].

Cognitive impairments are common among glioma patients, even in the absence of apparent neurological deficits. Neurological impairments can affect a wide range of domains, including attention, memory, executive function, and visuospatial processing, often with a substantial impact on patients’ quality of life and functional independence. Notably, a significant discrepancy frequently exists between patients’ subjective perception of their cognitive function and objective findings on formal neuropsychological testing, complicating clinical evaluation and decision-making [2,3].

Assessing neurocognitive performance in this population presents inherent challenges. Cognition encompasses a wide array of functions—ranging from perception and attention to language and executive control—which depend on distributed, large-scale brain networks that often lack a precise anatomical correlate [4,5]. These networks also exhibit substantial interindividual variability, further complicating efforts to localize and preserve function during surgical planning.

Previous studies have shown that more than 75% of glioma patients exhibit deficits in at least one cognitive domain upon formal testing [6,7]. These impairments can result from various factors, including tumor infiltration, peritumoral edema, tumor-related epilepsy, surgical resection, adjuvant therapy, and individual patient characteristics.

Left-hemispheric lesions—particularly those affecting language networks—frequently undergo awake craniotomy and intraoperative mapping. Right-hemispheric tumors are less commonly explored with the equivalent functional assessment, despite increasing evidence linking them to clinically meaningful deficits in higher-order brain functions, especially covering the cognitive domains of attention, visuospatial abilities, and executive functioning [1,8].

These are reliant on distributed functional networks and intact white-matter tracts, cannot be narrowed to localized brain areas, and have a major impact on the patient’s quality of life. Despite their high clinical relevance, cognitive impairments frequently remain underdiagnosed in the preoperative setting. To address this issue, the present study aimed to apply a structured domain-specific assessment to neurocognitive performance in patients with right-hemispheric gliomas over time and to correlate test results with the structural involvement of key white matter pathways, which were identified by non-invasive brain mapping and function-based tractography.

## 2. Materials and Methods

### 2.1. Patient Enrollment

Eighteen right-handed patients were prospectively enrolled between January 2024 and January 2025 in this observational study. Inclusion criteria were (1) age over 18 years, (2) fluency in the German language, (3) availability of preoperative navigated transcranial magnetic stimulation (nTMS) mapping and function-based fiber tracking, (4) histopathologically confirmed diagnosis of glioma, and (5) the completion of structured cognitive testing at all three time points—preoperatively, postoperatively, and at follow-up. Exclusion criteria comprised any contraindications to MRI scanning or nTMS mapping.

### 2.2. Cognitive Testing

#### 2.2.1. Task Overview

All patients underwent structured neuropsychological testing both preoperatively and postoperatively, as well as at a 3-month follow-up. The testing battery was selected to be easily applicable in clinical routine.

The Controlled Oral Word Association Test (COWAT) is a commonly used test of verbal fluency, with high reliability in assessing an individual’s cognitive processes, such as language retrieval and executive functioning [9,10]. The primary outcome is the total number of correct words generated for each letter within a given time limit [9,10].

The Montreal Cognitive Assessment (MoCA) was developed as a screening tool to detect mild cognitive impairment in patients and shows high test–retest reliability and excellent internal consistency [11,12]. The test consists of individual tasks that cover the domains of visuospatial/executive function, naming, memory, attention, language, abstraction, computation, and orientation [11,12].

The Hopkins Verbal Learning Test-Revised (HVLT-R) is a brief, standardized assessment designed to evaluate verbal learning efficiency, immediate and delayed recall, and recognition memory [13,14]. The test consists of a list of 12 nouns, with four words from each of three semantic categories, including three repeated learning trials and a delayed recall after 20–25 min [13,14].

The Digit Symbol Substitution Test (DSST) is a widely used neuropsychological assessment that measures processing speed and working memory [15,16]. It originates from the Wechsler Adult Intelligence Scale and consists of a task of pairing numbers with unique symbols [15,16,17].

The Trail Making Test is split into Part A (TMT-A) and Part B (TMT-B). The TMT-A consists of the task of connecting numbered circles in sequential order as quickly as possible, primarily assessing processing speed. On the contrary, the TMT-B reflects the ability to direct attention to changing stimuli and is assigned to the domain of attention and executive function, as circles have to be connected by alternating between numbers and letters in order [18,19]. The total task duration is measured for TMT-A and TMT-B [18,19].

The Bells Test is a cancellation task that requires participants to cross out bell icons randomly distributed among distractor images [20,21]. Three test parameters are analyzed. The Bells Test task completion time (BT-t) replicates the patient’s processing speed [20,21]. The Bells Test’s accuracy score (BT-AC) describes the number of bells correctly circled [20,21]. In addition, the Bells Test’s asymmetry score (BT-AS) is calculated from the difference in omissions between the left and right columns [20,21]. The BT-AC and BT-AS are both assigned to visuospatial functioning [20,21].

The Nine-Hole Peg Test (NHPT) is applied to measure finger dexterity and fine motor coordination, particularly of the upper extremities [22]. The test’s primary outcome measure is the time required to place and remove all nine pegs on a board with nine holes [22].

#### 2.2.2. Interpretation of Cognitive Results

All individual test scores were rescaled into z-scores (standard equivalents) using normative study data from published external norms on matching control groups for each patient for each test.

To determine the percentage of patients with impaired test results, we counted the number of individual patients with poor performance, defined as a performance worse than ≥1.0 SD of the normative data.

Group analysis considered test scores of all patients and mean group scores for individual tests, with an SD of 1.65 set to define an overall impaired group task performance, indicating that over 95% of the reference cohort performed better than the cohort of this study [23].

### 2.3. Function-Based Analysis of Structural Imaging

We conducted a function-based fiber tracking in all cases included in this study. Therefore, eloquent brain areas were identified non-invasively using navigated transcranial magnetic stimulation (nTMS) with a Nexstim eXimia NBS 5.2 device (Nexstim Plc, Helsinki, Finland). For testing, a verbal semantic association task, the Pyramids and Palm Trees Test (PPTT), was selected [24]. Stimulation sites were predefined using an established cortical parcellation system, which included 21 brain regions with 46 stimulation sites on each hemisphere, according to clinical routine [25]. A stimulation site was classified as positive if any kind of task error occurred; a differentiation in error category was not performed in these cases to capture the whole cortical network related to the PPTT task.

**Figure 1:**
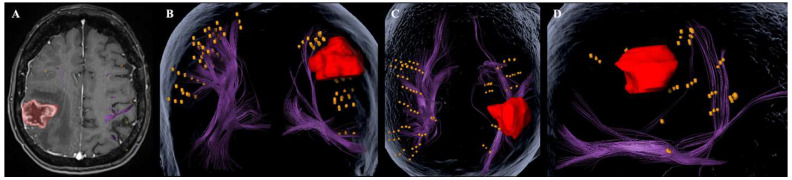
Illustrative case. This figure illustrates a representative case of a patient with a high-grade glioma located in the superior parietal lobe, demonstrating extensive disruption of the right-hemispheric network—most notably involving the superior longitudinal fasciculi I–III—and marked impairment of cognitive performance. Panel (**A**) shows anatomical imaging in the axial plane. Panels (**B**,**C**) present the three-dimensional bihemispheric network (purple) in coronal and axial views, respectively. Panel (**D**) illustrates the right-hemispheric network in a sagittal view. The tumor is shown in red, and nTMS-derived positive cortical stimulation sites are highlighted in orange.

nTMS-based cortical data were then used as regions of interest for function-based fiber tracking, using diffusion tensor imaging (DTI) b 1000 s/mm^2^, 30 directions, slice thickness 2 mm) (Figure 1). A single experienced rater performed tractography following an established institutional protocol to minimize inter-operator variability. Deterministic fiber tracking was performed with a minimum fiber length of 100 mm and a maximum angulation of 20° [26]. For fractional anisotropy (FA), we performed fiber tracking according to clinical routine at a fractional anisotropy threshold of 50% of the maximum FA [26].

### 2.4. Analysis of Imaging and Functional Status

Anatomical imaging was used to define the tumor location and calculate its volume. Additionally, network properties derived by function-based fiber tracking were assessed. The superior longitudinal fasciculi (SLF I, SLF II, SLF III), arcuate fasciculus (AF), inferior fronto-occipital fasciculus (IFOF), inferior longitudinal fasciculus (ILF), and uncinate fasciculus (UF) were analyzed with respect to tumor involvement (Figure 1). A fiber tract was considered affected if the tumor margins, excluding tumor-related edema, involved the fiber tract or the fiber tract could not be successfully reconstructed with the tumor located in the assumed course of the fiber tract. These findings were then correlated with individual neurocognitive test performance.

### 2.5. Statistical Analysis

Nonparametric statistical tests were applied, as several cognitive test variables failed normality testing (Shapiro–Wilk) and subgroup comparisons involved small, unbalanced sample sizes. Between-group comparisons were performed using the two-tailed Mann–Whitney U test, while paired comparisons of preoperative to postoperative and preoperative to follow-up test results were analyzed using the Wilcoxon signed-rank test.

To address the large number of comparisons across cognitive tests and fiber tracts, *p*-values were additionally corrected using the Benjamini–Hochberg false discovery rate procedure with a predefined q-value of 0.05. Only corrected *p*-values are shown in this manuscript. Effect size estimates were calculated for all Mann–Whitney U tests using the rank-biserial correlation (r) to evaluate the magnitude of observed effects independent of sample size.

All data sets were complete. All tests were two-tailed with a level of significance set at *p* < 0.05. Statistical analyses were conducted using GraphPad Prism (version 10.2 for Mac, GraphPad Software, La Jolla, CA, USA).

## 3. Results

### 3.1. General Data

Eighteen patients, aged 52.7 ± 18.3 years, with histopathologically confirmed supratentorial gliomas of the right hemisphere, were included in this study (Table 1). Tumor grading according to the WHO CNS classification revealed that the majority of cases were high-grade gliomas (Table 1). Tumor location was predominantly temporal (39%) and frontal (28%) (Table 1). Mean tumor volume was 33.0 ± 20.5 cm^3^.

### 3.2. Neurocognitive Testing

Regarding individual task performance, 16 patients (88.8%) showed impairment in at least one domain preoperatively, with one patient showing impairments in 9 of 10 tests, and 14 patients (77.8%) showed impairments at follow-up. We observed a high rate of preoperative cognitive impairments >1 SD, most notably in the BT-t, BT-AC and the COWAT (Table 2). Despite these impairments, postoperative changes were most prominent for the BT-t, BT-AC, and the COWAT; however, no significant surgery-related cognitive deterioration was observed. At follow-up, task performance improved across all tests, except for the BT-AS, with substantial reductions in patients with impairments of 38.9% for BT-t and 33.3% for TMT-B (Table 2).

Overall, the BT-t emerged as the most sensitive measure of impairments in this cohort, with the highest preoperative impairment rate (61.1%), most extensive deterioration postoperatively (16.7%), and the most significant reduction in impairments at follow-up (−38.9%).

Regarding cognitive domains, the most significant preoperative impairments were observed in attention and executive function (as measured by the TMT-B) and coordination (as measured by the NHPT) (Table 2). Regarding the cohort, as captured by z-scores, the greatest postoperative deterioration was observed for the NHPT with an increase in z-score from 1.21 ± 1.40 to 2.37 ± 2.24 (*p* = 0.0123), indicating worsened fine motor coordination immediately after surgery. However, performance partially recovered by follow-up (1.27 ± 2.01). Attention and executive function (TMT-B) showed the largest improvement at follow-up from a preoperative z-score of 1.49 ± 2.86 to 0.62 ± 2.87 (*p* = 0.333).

Other domains, such as language function (COWAT), episodic memory (HVLTR), global cognition (MoCA), and visuospatial function (BT-AC, BT-AS), remained stable over time, with no significant changes observed either postoperatively or at follow-up.

### 3.3. Tumor Location

Temporal lobe tumors were associated with relatively preserved cognitive performance, with all test results except the TMT-A remaining within normal limits, suggesting minor or no impairment (Table 3). Frontal brain tumors showed mild impairments regarding NHPT (1.60 ± 0.36), TMT-A (1.38 ± 0.81), and BT-t (1.29 ± 0.57) (Table 3).

Two patients in this study, suffering from parietal lobe tumors, exhibited the most severe and widespread cognitive impairments with clinically relevant impairments in TMT-A (3.98 ± 0.42), TMT-B (4.37 ± 1.97), NHPT (3.08 ± 1.85), BT-t (2.28 ± 1.91), and BT-AC (−5.18 ± 1.85).

### 3.4. Tumor Grading

Comparing tumor grades according to the World Health Organization Classification of Tumors of the Central Nervous System (WHO CNS°) [27], we did not observe statistically significant differences in test performance between low-grade glioma (LGG) (WHO CNS°1 and 2) and high-grade glioma (HGG) (WHO CNS°3 and 4) (Table 4). However, HGG overall performed worse, with impaired group task performance for TMT-A (1.94 ± 1.88), TMT-B (1.89 ± 3.2), and Bells-AC (−1.91 ± 2.13), particularly in the patients with parietal lesions, who both suffered from HGG.

### 3.5. Preoperative Mapping and Function-Based Fiber Tracking

**Figure 2:**
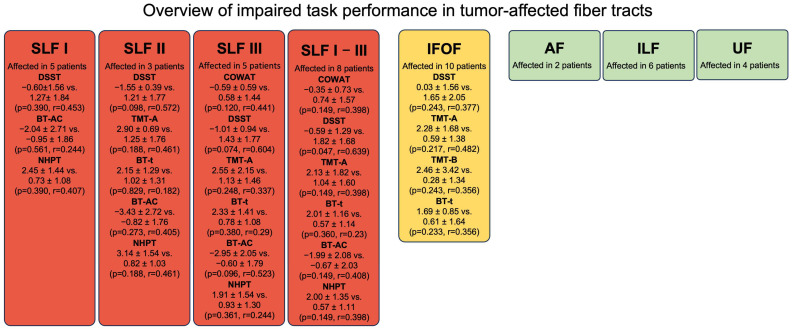
Overview of impaired task performance in tumor-affected fiber tracts. Tasks in which patients with tumor-affected right-hemispheric fiber tracts performed ≥1 standard deviation worse compared to patients without tract involvement are illustrated. SLF I/II/III—Superior Longitudinal Fasciculus I/II/III; AF—Arcuate Fasciculus; IFOF—Inferior Fronto-Occipital Fasciculus; ILF—Inferior Longitudinal Fasciculus; UF—Uncinate Fasciculus; COWAT—Controlled Oral Word Association Test; DSST—Digit Symbol Substitution Test; TMT-A—Trail Making Test Part A; BT-t—Bells Test task completion time; TMT-B—Trail Making Test Part B; NHPT—Nine-Hole Peg Test.

The right hemispheric network identified by function-based fiber tracking yielded a mean volume of 41.8 ± 22.7 cm^3^. Involvement of fiber tracts was observed most frequently for the IFOF (55.6%) and SLF I-III (44.4%) (Figure 2).

Patients with tumor involvement of the SLF I demonstrated impairments in the TMT-A (2.07 ± 1.25), BT-t (1.93 ± 0.983), BT-AC (−2.04 ± 2.71), and NHPT (2.45 ± 1.44).

Involvement of the SLF II was associated with more pronounced impairments in the TMT A (2.9 ± 0.69), BT-t (2.15 ± 1.29), BT-AC (−3.43 ± 2.72), and NHPT (3.14 ± 1.54) (Figure 2).

Patients with tumors affecting the SLF III showed clinically relevant impairments for TMT-A (2.55 ± 2.15), BT-t (2.33 ± 1.41), BT-AC (−2.95 ± 2.05) and NHPT (1.91 ± 1.54) (Figure 2).

When analyzing all SLF I–III affections combined, clinically relevant impaired performances were found for TMT-A (2.13 ± 1.82), BT-time (2.01 ± 1.16), BT-AC (−1.99 ± 2.08), and NHPT (2.0 ± 1.35), as well as a significantly worse performance in the DSST (−0.588 ± 1.29 vs. 1.82 ± 1.68, *p* = 0.047) (Figure 2).

Tumors affecting the IFOF were associated with a clinically relevant impaired performance in the TMT-A (2.28 ± 1.68), TMT-B (2.46 ± 3.42) and BT-time (1.69 ± 0.853) (Figure 2).

Affection of the AF (11.1% of cases), ILF (33.3% of cases), and the UF (22.2% of cases) was not associated with cognitive impairments across the tests performed.

## 4. Discussion

### 4.1. Summary of Results

In this cohort of right hemispheric glioma patients, we observed notable impairments in neuropsychological task performance, particularly in domains of processing speed, visuospatial attention, executive function, and fine motor coordination. The highest rates of preoperative cognitive impairments were observed at the individual patient level for the NHPT (66.7%), the Bells Test time (61.1%), and the Trail Making Tests A and B (TMT-A: 50.0%; TMT-B: 44.4%). Function-based tractography revealed that disruptions to the SLF complex and the IFOF were associated with impaired task performance.

### 4.2. Task Selection

Within the task battery employed in this study, the Bells Test time emerged as the most sensitive measure for detecting cognitive impairments in patients with right hemispheric lesions. It demonstrated the highest preoperative impairment rate (61.1%), the highest postoperative deterioration (16.7%), and the most substantial reduction in impairments at follow-up (−38.9%). In line with this, the NHPT, TMT-A, and TMT-B revealed the highest preoperative impairment rates, highlighting their utility as brief and sensitive screening tools in this population, similar to previous literature [28,29]. Notably, all four tasks can be administered within a total testing time of less than 10 min, supporting their feasibility in clinical settings. Conversely, fewer than 15% of patients exhibited preoperative impairments on the COWAT, HVLTR, and MoCA. These tests, targeting language function, episodic memory, and global cognition, respectively, may be less sensitive to right-hemispheric dysfunction or may suffer from limited specificity in this context. Additionally, the MoCA may lack discriminatory power in this cohort due to its broad scope or the use of normative data not optimally tailored to this patient population.

### 4.3. Structure-Function Correlation

Although eloquence is mainly associated with primary motor areas and left-hemispheric language areas, evidence from functional imaging, stroke patients, and tumor resection demonstrates that the right hemisphere plays an essential role in visuospatial attention, attention and executive function, sensory integration, and social cognition [8,30,31].

The observed associations between tract involvement and neurocognitive deficits in this study align with disruptions of large-scale networks typically anchored in the right hemisphere [8].

Frontoparietal networks such as the dorsal attention network and the frontoparietal control network rely on the integrity of SLF I–III subdivisions, which exhibit distinct functional specializations [32]. The SLF I links dorsal attention regions and is associated with spatial orientation and executive attention, whereas SLF III couples inferior frontal regions with the temporoparietal junction and allows for sensorimotor–attentional coupling and stimulus reorienting [8]. The SLF II integrates parietal and frontal association nodes, allowing for interaction between these systems [8]. This aligns with the observed impairments in task performance of the TMT-A, BT-t, and DSST for processing speed and the BT-AC for visuospatial attention [33]. These findings are consistent with prior work highlighting the role of the SLF network in attentional and spatial processing [33].

The IFOF connects frontal areas with occipital, parietal, and temporal regions [34,35]. In the left hemisphere, the IFOF is associated with semantic language processing and executive control [34,35]. In the right hemisphere, this tract is implicated in attention, spatial cognition, face perception, nonverbal semantic cognition, and emotion regulation [34,35,36]. Patients with IFOF involvement demonstrated impairments in TMT-A and Bells Test time for processing speed, TMT-B for attention and executive function, and HVLT, potentially reflecting deficits in nonverbal semantic processing, such as categorical retrieval.

The inferior longitudinal fasciculus is a long-range fiber tract that primarily connects the occipital lobe with the anterior temporal lobe and has been associated with face processing in the right hemisphere, as well as aspects of multimodal semantic integration [37]. However, unilateral anterior ILF disruption is often clinically compensated. Patients with focal damage to the anterior temporal lobe—including vascular lesions or surgical resections—typically exhibit only mild to moderate semantic impairment, despite this region’s recognized role in semantic cognition [38]. This has been attributed to the bilateral organization of semantic networks, which renders the system more resilient to unilateral insult [38,39]. Moreover, ILF-mediated functions may be dynamically supported by alternative pathways, particularly in diffuse low-grade glioma, where efficient neuroplastic reorganization and distributed compensation have been documented [40]. These mechanisms likely contribute to the relative sparing we observed in patients with right temporal involvement.

The arcuate fasciculus is a dorsal perisylvian association tract, connecting posterior regions of the superior and middle temporal gyrus to the perisylvian parietal and frontal cortex [41]. In the right hemisphere, the AF has been implicated in social cognition, music perception and spatial cognition rather than phonological language output [41]. In our cohort, only two patients exhibited tumor involvement of the right AF, and no associated impairments were detected on the applied cognitive battery. This observation likely reflects both the lower functional burden of the right AF compared with its left-hemispheric counterpart and the capacity of distributed right-hemisphere networks to compensate for impairments. It may also relate to the fact that specific right-sided functions of the AF, such as social cognition, were not directly assessed by the tests used in this study.

Moreover, the anatomical location of the tumor appeared to influence cognitive performance. Patients with parietal tumors showed a trend toward greater impairment, likely due to the proximity of these lesions to the temporoparietal junction and multiple critical association fibers.

### 4.4. Clinical Implications

These results emphasize the importance of expanding the concept of “eloquence” in neuro-oncology beyond traditional motor and language domains. Right hemispheric tumors, particularly those affecting frontoparietal regions, can produce significant impairments in attention and executive function, visuospatial attention, and processing speed—domains often underrepresented in routine clinical assessments.

To address this issue, awake resection of right-hemispheric gliomas with intraoperative mapping is a viable option, having been shown to preserve complex cognitive functions more effectively and support higher return-to-work rates compared to resections under general anesthesia, despite a similar extent of resection [1,42]. The use of intraoperative mapping techniques, particularly in patients with minimal or no preoperative deficits, enables more accurate identification and preservation of critical cognitive networks, thereby contributing to improved surgical outcomes and postoperative quality of life [1,42].

In terms of tumor location, we did not observe meaningful impairments induced by temporal lesions or those affecting the ILF in this study. This aligns with the current neuro-oncological practice for temporal glioblastoma, in which superior outcomes have been observed for temporal lobectomy compared to gross total resection [43].

A structured, domain-specific neuropsychological test is crucial for accurately identifying tumor-induced cognitive dysfunction. Brief bedside assessments may fail to detect subtle deficits, particularly in patients with high pre-morbid cognitive function. Incorporating function-based tractography into preoperative planning provides a valuable tool for identifying at-risk structures and guiding surgical strategy.

Diffuse gliomas induce cortical plasticity both pre- and postoperatively, with reorganization patterns occurring in perilesional regions, across large distributed networks within the affected hemisphere, and also involving the contralesional side [44]. These findings support the notion of a largely similar functional organization between the right and left hemispheres. In patients with left-sided gliomas, cortical plasticity through the recruitment of right-hemispheric regions has been demonstrated both before and after surgery [45,46,47]. More broadly, the right hemisphere contributes to higher-order language functions, as evidenced by right-hemispheric strokes that can result in deficits of these functions [48,49].

Most impairments were already present preoperatively, indicating a tumor-induced etiology. Postoperative improvements at follow-up suggest that surgical resection can not only preserve but also facilitate neurological recovery, likely through relief of mass effect and functional reorganization.

Taken together, these findings suggest reconsidering how eloquence and maximal safe resection should be conceptualized in right-hemispheric glioma surgery. Our data support a broader view on eloquence in which visuospatial attention, executive function, processing speed, and social-affective processes constitute equally relevant and eloquent functional domains. This reinterpretation has tangible consequences for operative strategy. Low rates of cognitive impairments in right temporal tumors support wider resection margins when white-matter boundaries are respected, consistent with the resilience of ILF-mediated functions in the nondominant hemisphere [43,50].

By contrast, deep-seated lesions within parietal regions or the temporo-parietal junction should be regarded as high-risk locations due to their integration within SLF-based attention networks. Accordingly, these cases warrant extended preoperative cognitive profiling, tract-based planning, and awake mapping in selected instances, despite their location in the nominally ‘nondominant’ hemisphere. Incorporating targeted cognitive testing and tract-specific analyses into the standard neurosurgical workflow may improve risk stratification, surgical planning, and ultimately patient outcomes [51].

### 4.5. Limitations

The primary limitation of this study is its small sample size of 18 patients, reflecting its exploratory nature as a prospective cohort. The small cohort size reduces statistical power, potentially leading to clinically meaningful differences failing to reach statistical significance while increasing the likelihood of Type II errors. Due to the heterogeneity of glioma location, variable tract involvement patterns, and cognitive profiles, an a priori power analysis was not feasible and would not have yielded reliable assumptions. Although we applied false discovery rate correction and reported effect sizes to improve interpretability, the findings of this study should be critically viewed as preliminary and hypothesis-generating.

Additionally, the brevity of the neuropsychological test battery may limit its sensitivity, and reliance on normative data from the literature may not fully align with the demographic characteristics of this study’s patient population. Furthermore, potential learning effects must be considered when interpreting longitudinal improvements in performance [52]. To mitigate this, alternate test versions (e.g., MoCA, HVLT-R) were administered at each assessment time point. Future studies with larger cohorts, applying multivariate statistical models to disentangle the effects of different clinical and demographic factors, are warranted to validate these findings and further to refine preoperative cognitive assessment protocols in glioma patients. The next step is to evaluate whether network-guided surgery, including preoperative and intraoperative mapping of higher cognitive functions, improves postoperative autonomy.

## 5. Conclusions

This study emphasizes the high prevalence and domain-specific patterns of cognitive impairments in patients with right hemispheric gliomas, particularly affecting processing speed, visuospatial attention, executive function, and fine motor coordination. Combining structured neuropsychological testing with function-based tractography offers a detailed understanding of tumor-induced network disruption and its clinical implications. Our findings highlight the importance of right-hemispheric association tracts, specifically the SLF I-III and IFOF, in cognitive functions that are often neglected in routine preoperative assessments.

## Figures and Tables

**Table 1:** Patient and tumor characteristics. This table outlines the patient and tumor characteristics, including histopathological findings according to the World Health Organization Classification of Tumors of the Central Nervous System (WHO CNS°) of all patients included in the study.

Number of Patients (n)	18
**Gender**	
Male	10 (56%)
Female	8 (44%)
**Age** (Mean ± SD)	52.7 ± 18.3 years
**Hemisphere** (n (%))	
Left	0 (0%)
Right	18 (100%)
**Localization** (n (%))	
Frontal	5 (28%)
Parietal	2 (11%)
Temporal	7 (39%)
Temporoinsular	1 (6%)
Frontoinsular	1 (6%)
Occipital	1 (6%)
Basal ganglia	1 (6%)
**WHO CNS degree** (n (%))	
°1	1 (6%)
°2	4 (22%)
°3	2 (11%)
°4	11 (61%)
**Tumor volume** (Mean ± SD)	33.0 ± 20.5 cm^3^
**Fiber volume** (Mean ± SD)	41.8 ± 22.7 cm^3^

**Table 2:** Impairments in neurocognitive tests sorted by cognitive domains. This table outlines test results as mean z-Scores and standard deviations (SD) for neurocognitive testing preoperatively (pre), postoperatively (post), and at follow-up (FU). COWAT—Controlled Oral Word Association Test; HVLTR—Hopkins Verbal Learning Test—Revised; DSST—Digit Symbol Substitution Test; TMT-A—Trail Making Test Part A; BT-t—Bells Test task completion time; TMT-B—Trail Making Test Part B; BT-AC—Bells Test’s accuracy score; BT-AS—Bells Test’s asymmetry score NHPT—Nine-Hole Peg Test; MoCA—Montreal Cognitive Assessment.

Domain	Neuro-Cognitive Test	Preoperative z-Scores Mean (SD)	Postoperative z-Scores Mean (SD)	*p*-Value Pre-Post	Follow-Up z-Scores Mean (SD)	*p*-Value Pre-FU
**Language**	**COWAT**	0.26 ± 1.35	0.04 ± 0.91	0.871	0.35 ± 1.66	0.772
**Episodic memory**	**HVLTR**	0.01 ± 0.80	−0.16 ± 0.82	0.339	0.27 ± 0.61	0.744
**Processing speed**	**DSST**	0.75 ± 1.92	0.32 ± 1.87	0.339	0.59 ± 2.14	0.744
	**TMT-A**	1.53 ± 1.74	**2.13 ± 2.47**	0.339	1.14 ± 1.94	0.744
	**BT-t**	1.21 ± 1.34	1.39 ± 1.24	0.405	0.45 ± 1.25	0.333
**Attention and executive function**	**TMT-B**	1.49 ± 2.86	**1.95 ± 2.82**	0.339	0.62 ± 2.87	0.333
**Visuospatial function**	**BT-AC**	−1.25 ± 2.10	−1.18 ± 2.99	0.904	−1.04 ± 2.30	0.744
	**BT-AS**	0.01 ± 1.63	0.79 ± 2.23	0.339	−0.24 ± 1.47	0.744
**Coordination**	**NHPT**	1.21 ± 1.40	**2.37 ± 2.24**	0.123	1.27 ± 2.01	0.744
**Screening**	**MoCA**	0.54 ± 1.06	0.48 ± 1.26	0.947	0.46 ± 1.15	0.744

**Table 3:** Correlation of tumor location with test performance. This table correlates tumor location with preoperative test performance, shown as mean z-Scores and standard deviations (SD). COWAT—Controlled Oral Word Association Test; HVLTR—Hopkins Verbal Learning Test—Revised; DSST—Digit Symbol Substitution Test; TMT-A—Trail Making Test Part A; BT-t—Bells Test task completion time; TMT-B—Trail Making Test Part B; BT-AC—Bells Test’s accuracy score; BT-AS—Bells Test’s asymmetry score; NHPT—Nine-Hole Peg Test; MoCA—Montreal Cognitive Assessment.

	Patients (n)	COWAT	MOCA	DSST	HVLT	TMT-A	TMT-B	BT-t	BT-AC	BT-AS	NHPT
**temporal**	7	0.82 ± 1.96	0.88 ± 1.15	1.42 ± 2.06	−0.07 ± 1.05	1.21 ± 2.08	0.68 ± 1.50	0.89 ± 1.26	−0.97 ± 2.06	−1.08 ± 1.78	0.44 ± 0.91
**frontal**	5	−0.24 ± 0.41	0.97 ± 0.92	0.13 ± 1.34	−0.06 ± 0.60	1.38 ± 0.81	0.84 ± 1.42	1.29 ± 0.57	−0.60 ± 1.31	0.66 ± 0.59	1.60 ± 0.36
**parietal**	2	−0.89 ± 0.31	−0.44 ± 0.52	−1.25 ± 1.05	−0.11 ± 0.40	**3.98 ± 0.42**	**4.37 ± 1.97**	**2.28 ± 1.91**	**−5.18 ± 1.85**	0.55 ± 0.76	**3.08 ± 1.85**
**insular**	2	0.09 ± 0.77	−0.26 ± 0.79	**1.99 ± 2.28**	0.30 ± 1.38	−0.38 ± 1.00	−0.58 ± 1.12	1.09 ± 3.60	−0.93 ± 1.39	1.10 ± 3.08	−0.40 ± 1.03
**occipital**	1	0.85 ± 0.00	1.04 ± 0.00	−1.25 ± 0.00	0.72 ± 0.00	**2.63 ± 0.00**	−0.01 ± 0.00	1.28 ± 0.00	−1.25 ± 0.00	1.09 ± 0.00	**3.61 ± 0.00**
**basal ganglia**	1	0.85 ± 0.00	−0.81 ± 0.00	**2.72 ± 0.00**	−0.11 ± 0.00	2.21 ± 0.00	**10.30 ± 0.00**	1.15 ± 0.00	0.71 ± 0.00	0.01 ± 0.00	**1.70 ± 0.00**

**Table 4:** Correlation of tumor grading with test performance. This table correlates tumor grading, divided into low-grade glioma (LGG) and high-grade glioma (HGG), with preoperative test performance, illustrated as mean z-Scores and standard deviations. COWAT—Controlled Oral Word Association Test; HVLTR—Hopkins Verbal Learning Test—Revised; DSST—Digit Symbol Substitution Test; TMT-A—Trail Making Test Part A; BT-t—Bells Test task completion time; TMT-B—Trail Making Test Part B; BT-AC—Bells Test’s accuracy score; BT-AS—Bells Test’s asymmetry score; NHPT—Nine-Hole Peg Test; MoCA—Montreal Cognitive Assessment.

	Patients (n)	COWAT	MOCA	DSST	HVLT	TMT-A	TMT-B	BT-t	BT-AC	BT-AS	NHPT
**LGG**	5	−0.108 ± 0.664	1.04 ± 0.906	1.57 ± 1.25	−0.17 ± 1.2	0.446 ± 0.433	0.45 ± 1.47	1.48 ± 0.622	0.446 ± 0.361	0.442 ± 0.592	0.59 ± 1.02
**HGG**	13	0.397 ± 1.54	0.356 ± 1.09	0.436 ± 2.08	0.08 ± 0.636	**1.94 ± 1.88**	**1.89 ± 3.2**	1.11 ± 1.54	**−1.91 ± 2.13**	−0.162 ± 1.88	1.44 ± 1.48
***p*** **-value**		0.706	0.434	0.434	0.853	0.434	0.537	0.537	0.189	0.706	0.434

## Data Availability

In the interest of patient privacy, all the collected raw data of individual cases are not publicly available. The anonymous datasets used and analyzed during the current study, the study protocol, and the statistical analysis plan are available upon reasonable request from the corresponding author to researchers who provide a methodologically sound proposal beginning 3 months and ending 5 years following article publication. Proposals should be directed to maximilian.schwendner@med.uni-heidelberg.de; to gain access, data requestors will need to sign a data access agreement.

## Abbreviations

The following abbreviations are used in this manuscript:

AF	Arcuate fasciculus
BT-AC	Bells Test accuracy score
BT-AS	Bells Test asymmetry score
BT-t	Bells Tes task completion time
COWAT	Controlled oral word association test
DSST	Digit symbol substitution test
FU	Follow-up
HGG	High-grade glioma
HVLTR	Hopkins verbal learning test revised
IFOF	Inferior fronto-occipital fasciculus
ILF	Inferior longitudinal fasciculus
LGG	Low-grade glioma
MoCA	Montreal cognitive assessment
MRI	Magnetic resonance imaging
NHPT	Nine-hole peg test
nTMS	Navigated transcranial magnetic stimulation
SLF I	Superior longitudinal fasciculus I
SLF II	Superior longitudinal fasciculus II
SLF III	Superior longitudinal fasciculus III
TMT-A	Trail making test part A
TMT-B	Trail making test part B
UF	Uncinate fasciculus
WHO CNS	Tumor grades according to the World Health Organization Classification of Tumors of the Central Nervous System

## References

[1] Ramirez-Ferrer E., Aguilera-Pena M.P., Duffau H. (2024). Functional and oncological outcomes after right hemisphere glioma resection in awake versus asleep patients: A systematic review and meta-analysis. Neurosurg. Rev..

[2] Gehring K., Taphoorn M.J., Sitskoorn M.M., Aaronson N.K., Dirven L., Armstrong T.S. (2015). Predictors of subjective versus objective cognitive functioning in patients with stable grades II and III glioma. Neuro-Oncol. Pract..

[3] Pertz M., Kowalski T., Jetschke K., Schmieder K., Schlegel U., Miller D. (2022). Pre- and postoperative self-reported and objectively assessed neurocognitive functioning in lower grade glioma patients. J. Clin. Neurosci..

[4] Fuster J.M. (2006). The cognit: A network model of cortical representation. Int. J. Psychophysiol..

[5] Kanai R., Rees G. (2011). The structural basis of inter-individual differences in human behaviour and cognition. Nat. Rev. Neurosci..

[6] Habets E.J.J., Kloet A., Walchenbach R., Vecht C.J., Klein M., Taphoorn M.J.B. (2014). Tumour and surgery effects on cognitive functioning in high-grade glioma patients. Acta Neurochir..

[7] Tucha O., Smely C., Preier M., Lange K.W. (2000). Cognitive deficits before treatment among patients with brain tumors. Neurosurgery.

[8] Bernard F., Lemee J.M., Ter Minassian A., Menei P. (2018). Right Hemisphere Cognitive Functions: From Clinical and Anatomic Bases to Brain Mapping During Awake Craniotomy Part I: Clinical and Functional Anatomy. World Neurosurg..

[9] Zhou A., Britt C., Woods R.L., Orchard S.G., Murray A.M., Shah R.C., Rajan R., McNeil J.J., Chong T.T.-J., Storey E. (2023). Normative Data for Single-Letter Controlled Oral Word Association Test in Older White Australians and Americans, African-Americans, and Hispanic/Latinos. J. Alzheimer’s Dis. Rep..

[10] Villalobos D., Torres-Simón L., Pacios J., Paúl N., del Río D. (2023). A Systematic Review of Normative Data for Verbal Fluency Test in Different Languages. Neuropsychol. Rev..

[11] Nasreddine Z.S., Phillips N.A., Bédirian V., Charbonneau S., Whitehead V., Collin I., Cummings J.L., Chertkow H. (2005). The Montreal Cognitive Assessment, MoCA: A brief screening tool for mild cognitive impairment. J. Am. Geriatr. Soc..

[12] Thomann A.E., Goettel N., Monsch R.J., Berres M., Jahn T., Steiner L.A., Monsch A.U. (2018). The Montreal Cognitive Assessment: Normative Data from a German-Speaking Cohort and Comparison with International Normative Samples. J. Alzheimer’s Dis..

[13] Duff K. (2015). Demographically corrected normative data for the Hopkins Verbal Learning Test-Revised and Brief Visuospatial Memory Test-Revised in an elderly sample. Appl. Neuropsychol. Adult.

[14] Ryan J., Woods R.L., Murray A.M., Shah R.C., Britt C.J., Reid C.M., Wolfe R., Nelson M.R., Lockery J.E., Orchard S.G. (2021). Normative performance of older individuals on the Hopkins Verbal Learning Test-Revised (HVLT-R) according to ethno-racial group, gender, age and education level. Clin. Neuropsychol..

[15] Ryan J.J., Woods R.L., Britt C.J., Murray A.M., Shah R.C., Reid C.M., Wolfe R., Nelson M.R., Orchard S.G., Lockery J.E. (2020). Normative Data for the Symbol Digit Modalities Test in Older White Australians and Americans, African-Americans, and Hispanic/Latinos. J. Alzheimer’s Dis. Rep..

[16] Jaeger J. (2018). Digit Symbol Substitution Test: The Case for Sensitivity Over Specificity in Neuropsychological Testing. J. Clin. Psychopharmacol..

[17] Wechsler D. (2008). Wechsler Adult Intelligence Scale—Fourth Edition (WAIS-IV).

[18] Tombaugh T. (2004). Trail Making Test A and B: Normative data stratified by age and education. Arch. Clin. Neuropsychol..

[19] Reitan R.M. (1955). The relation of the trail making test to organic brain damage. J. Consult. Psychol..

[20] Mancuso M., Damora A., Abbruzzese L., Navarrete E., Basagni B., Galardi G., Caputo M., Bartalini B., Bartolo M., Zucchella C. (2018). A New Standardization of the Bells Test: An Italian Multi-Center Normative Study. Front. Psychol..

[21] Gauthier L., Dehaut F., Joanette Y. (1989). The Bells Test: A quantitative and qualitative test for visual neglect. Int. J. Clin. Neuropsychol..

[22] Grice K.O., Vogel K.A., Le V., Mitchell A., Muniz S., Vollmer M.A. (2003). Adult norms for a commercially available Nine Hole Peg Test for finger dexterity. Am. J. Occup. Ther..

[23] Ng S., Rigau V., Moritz-Gasser S., Gozé C., Darlix A., Herbet G., Duffau H. (2024). Long-term autonomy, professional activities, cognition, and overall survival after awake functional-based surgery in patients with IDH-mutant grade 2 gliomas: A retrospective cohort study. Lancet Reg. Health Eur..

[24] Howard D., Patterson K.E. (1992). The Pyramids and Palm Trees Test.

[25] Corina D.P., Gibson E.K., Martin R., Poliakov A., Brinkley J., Ojemann G.A. (2005). Dissociation of action and object naming: Evidence from cortical stimulation mapping. Hum. Brain Mapp..

[26] Sollmann N., Meyer B., Krieg S.M. (2017). Implementing Functional Preoperative Mapping in the Clinical Routine of a Neurosurgical Department: Technical Note. World Neurosurg..

[27] Louis D.N., Perry A., Wesseling P., Brat D.J., Cree I.A., Figarella-Branger D., Hawkins C., Ng H.K., Pfister S.M., Reifenberger G. (2021). The 2021 WHO Classification of Tumors of the Central Nervous System: A summary. Neuro Oncol..

[28] Dwan T.M., Ownsworth T., Chambers S., Walker D.G., Shum D.H.K. (2015). Neuropsychological assessment of individuals with brain tumor: Comparison of approaches used in the classification of impairment. Front. Oncol..

[29] Ng S., Herbet G., Lemaitre A.-L., Cochereau J., Moritz-Gasser S., Duffau H. (2020). Neuropsychological assessments before and after awake surgery for incidental low-grade gliomas. J. Neurosurg..

[30] Hartwigsen G., Bengio Y., Bzdok D. (2021). How does hemispheric specialization contribute to human-defining cognition?. Neuron..

[31] Hartikainen K.M. (2021). Emotion-Attention Interaction in the Right Hemisphere. Brain Sci..

[32] Kamali A., Flanders A.E., Brody J., Hunter J.V., Hasan K.M. (2014). Tracing superior longitudinal fasciculus connectivity in the human brain using high resolution diffusion tensor tractography. Brain Struct. Funct..

[33] Janelle F., Iorio-Morin C., D’Amour S., Fortin D. (2022). Superior Longitudinal Fasciculus: A Review of the Anatomical Descriptions With Functional Correlates. Front. Neurol..

[34] Giampiccolo D., Herbet G., Duffau H. (2025). The inferior fronto-occipital fasciculus: Bridging phylogeny, ontogeny and functional anatomy. Brain.

[35] Alam T.R.J.G., Arias J.C., Jefferies E., Smallwood J., Leemans A., Davolos J.M. (2024). Ventral and dorsal aspects of the inferior frontal-occipital fasciculus support verbal semantic access and visually-guided behavioural control. Brain Struct. Funct..

[36] Herbet G., Moritz-Gasser S., Duffau H. (2017). Direct evidence for the contributive role of the right inferior fronto-occipital fasciculus in non-verbal semantic cognition. Brain Struct. Funct..

[37] Herbet G., Zemmoura I., Duffau H. (2018). Functional Anatomy of the Inferior Longitudinal Fasciculus: From Historical Reports to Current Hypotheses. Front. Neuroanat..

[38] Ralph M.A.L., Cipolotti L., Manes F., Patterson K. (2010). Taking both sides: Do unilateral anterior temporal lobe lesions disrupt semantic memory?. Brain..

[39] Rice G.E., Hoffman P., Ralph M.A.L. (2015). Graded specialization within and between the anterior temporal lobes. Ann. N. Y. Acad. Sci..

[40] Mandonnet E., Nouet A., Gatignol P., Capelle L., Duffau H. (2007). Does the left inferior longitudinal fasciculus play a role in language? A brain stimulation study. Brain.

[41] Vavassori L., Venturini M., Zigiotto L., Annicchiarico L., Corsini F., Avesani P., Petit L., De Benedictis A., Sarubbo S. (2023). The arcuate fasciculus: Combining structure and function into surgical considerations. Brain Behav..

[42] Mamadaliev D.M., Saito R., Motomura K., Ohka F., Scalia G., Umana G.E., Conti A., Chaurasia B. (2024). Awake Craniotomy for Gliomas in the Non-Dominant Right Hemisphere: A Comprehensive Review. Cancers.

[43] Arvaniti C.K., Karagianni M.D., Papageorgakopoulou M.A., Brotis A.G., Tasiou A., Fountas K.N. (2024). The role of lobectomy in glioblastoma management: A systematic review and meta-analysis. Brain Spine.

[44] Nieberlein L., Rampp S., Gussew A., Prell J., Hartwigsen G. (2023). Reorganization and Plasticity of the Language Network in Patients with Cerebral Gliomas. Neuroimage Clin..

[45] Zimmermann M., Rössler K., Kaltenhäuser M., Grummich P., Yang B., Buchfelder M., Doerfler A., Kölble K., Stadlbauer A. (2020). Refined Functional Magnetic Resonance Imaging and Magnetoencephalography Mapping Reveals Reorganization in Language-Relevant Areas of Lesioned Brains. World Neurosurg..

[46] Krieg S.M., Sollmann N., Hauck T., Ille S., Foerschler A., Meyer B., Ringel F. (2013). Functional language shift to the right hemisphere in patients with language-eloquent brain tumors. PLoS ONE.

[47] Ille S., Engel L., Albers L., Schroeder A., Kelm A., Meyer B., Krieg S.M. (2019). Functional Reorganization of Cortical Language Function in Glioma Patients-A Preliminary Study. Front. Oncol..

[48] Gajardo-Vidal A., Lorca-Puls D.L., Hope T.M.H., Jones O.P., Seghier M.L., Prejawa S., Crinion J.T., Leff A.P., Green D.W., Price C.J. (2018). How right hemisphere damage after stroke can impair speech comprehension. Brain.

[49] Lindell A.K. (2006). In your right mind: Right hemisphere contributions to language processing and production. Neuropsychol. Rev..

[50] Fava A., Lisi S.V., Mauro L., Morace R., Ciavarro M., Gorgoglione N., Petrella G., Quarato P.P., Di Gennaro G., di Russo P. (2024). The anterior sylvian point as a reliable landmark for the anterior temporal lobectomy in mesial temporal lobe epilepsy: Technical note, case series, and cadaveric dissection. Front. Med..

[51] Morshed R.A., Young J.S., Kroliczek A.A., Berger M.S., Brang D., Hervey-Jumper S.L. (2021). A Neurosurgeon’s Guide to Cognitive Dysfunction in Adult Glioma. Neurosurgery.

[52] Bartels C., Wegrzyn M., Wiedl A., Ackermann V., Ehrenreich H. (2010). Practice effects in healthy adults: A longitudinal study on frequent repetitive cognitive testing. BMC Neurosci..

